# First report of *kdr* mutations in the voltage-gated sodium channel gene in the arbovirus vector, *Aedes aegypti*, from Nouakchott, Mauritania

**DOI:** 10.1186/s13071-023-06066-8

**Published:** 2023-12-19

**Authors:** Mohamed Aly Ould Lemrabott, Sébastien Briolant, Nicolas Gomez, Leonardo Basco, Ali Ould Mohamed Salem Boukhary

**Affiliations:** 1 Université de Nouakchott, UR-GEMI, Nouveau Campus Universitaire, BP 5026, Nouakchott, Mauritania; 2 Aix Marseille Université, IRD, AP-HM, SSA, VITROME, Marseille, France; 3 IHU-Méditerranée Infection, Marseille, France; 4 Unité de Parasitologie Entomologie, Département de Microbiologie et Maladies Infectieuses, Institut de Recherche Biomédicale des Armées (IRBA), Marseille, France

**Keywords:** *Aedes aegypti*, Mauritania, Voltage-gated sodium channel, *kdr*, Point mutation, Pyrethroids

## Abstract

**Background:**

Since 2014, dengue epidemics have occurred almost annually in Nouakchott, the capital city of Mauritania, coinciding with the recent establishment of *Aedes aegypti*, the primary vector of dengue, in the city. *Anopheles arabiensis*, the primary vector of malaria, is also abundant not only in Nouakchott but also in most areas of the country. Resistance to insecticides has been studied in *An. arabiensis* but not in *Ae. aegypti* in Mauritania. The objective of the present study was to establish the baseline data on the frequencies of knockdown resistance (*kdr*) mutations in the voltage-gated sodium channel (*vgsc*) gene in *Ae. aegypti* collected in Nouakchott to improve vector control.

**Methods:**

Resting *Ae. aegypti* mosquitoes were collected in 2017 and 2018 in Teyarett and Dar Naim districts in Nouakchott using a battery-powered aspirator. Polymerase chain reaction (PCR) and DNA sequencing were performed to detect the presence of five *kdr* mutations known to be associated with pyrethroid resistance: L982W, S989P, I1011M/G, V1016G/I, and F1534C.

**Results:**

A total of 100 female *Ae. aegypti* mosquitoes were identified among collected resting culicid fauna, of which 60% (60/100) were unfed, 12% (12/100) freshly blood-fed, and 28% (28/100) gravid. Among the mutations investigated in this study, 989P, 1016G, and 1534C were found to be widespread, with the frequencies of 0.43, 0.44, and 0.55, respectively. Mutations were not found in codons 982 and 1011. No other mutations were detected within the fragments analyzed in this study. Genotype distribution did not deviate from Hardy–Weinberg equilibrium. The most frequent co-occurring point mutation patterns among *Ae. aegypti* mosquitoes were the heterozygous individuals 989SP/1016VG/1534FC detected in 45.1% of mosquitoes. In addition, homozygous mutant 1534CC co-occurred simultaneously with homozygous wild type 989SS and 1016VV in 30.5% of mosquito specimens. Inversely, homozygous wild-type 1534FF co-occurred simultaneously with homozygous mutant 989PP and 1016GG in 19.5% of the mosquitoes.

**Conclusions:**

To our knowledge, this is the first study reporting the presence of three point mutations in the *vgsc* gene of *Ae. aegypti* in Mauritania. The findings of the present study are alarming because they predict a high level of resistance to pyrethroid insecticides which are commonly used in vector control in the country. Therefore, further studies are urgently needed, in particular phenotypic characterization of insecticide resistance using the standardized test.

**Graphical Abstract:**

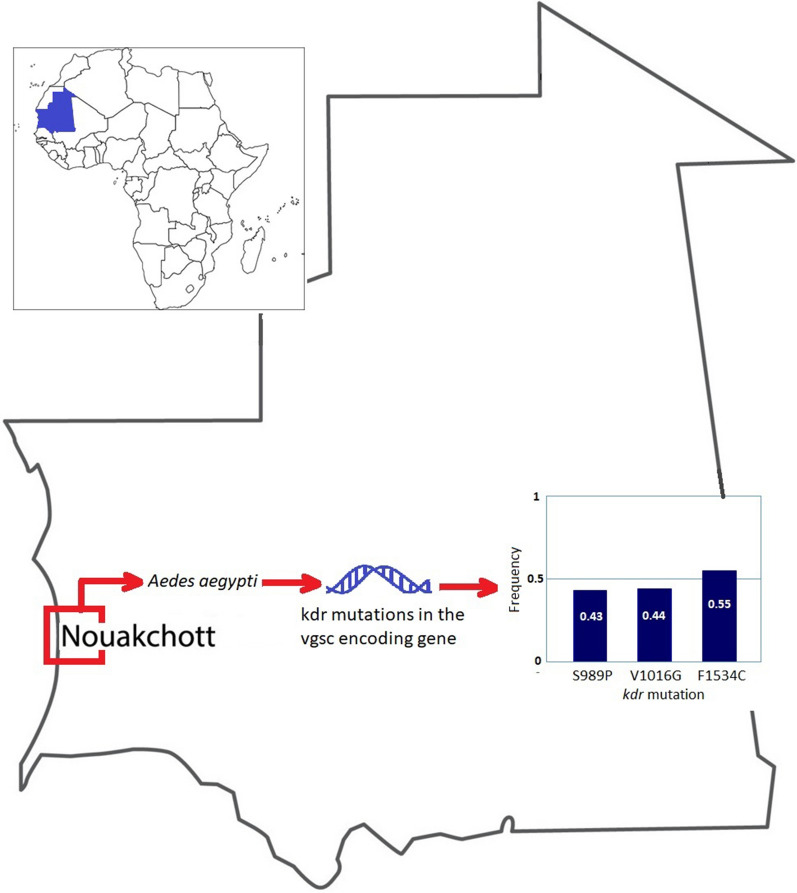

## Introduction

Dengue is the most widespread mosquito-borne viral disease in the world [1]. In Mauritania, dengue has also been a significant public health problem since the first documented dengue outbreak in 2014 in Nouakchott, the capital city [2, 3]. The emergence of dengue coincided with the first detection of *Aedes aegypti* in the city [4]. Since 2014, dengue cases and outbreaks have been reported annually in Nouakchott and other urban settings, particularly in the Oasian city of Atar and the mining city of Zouérat in the northern Saharan zone [5–7].

*Aedes aegypti* is the major vector of dengue as well as three other arboviruses of public health importance, namely yellow fever, chikungunya, and Zika [8]. This mosquito species is well adapted to thrive in urban environments where suitable human-made habitats for mosquito vectors exist and contact between mosquito vectors and human hosts is frequent, facilitating arbovirus transmission [9]. At present, there is no specific treatment for dengue. Only one licensed vaccine (Dengvaxia^®^) is currently available in several Asian and Latin American countries, but it is not recommended for the general population; it may be administered only in children (aged 9–16 years) with a previous history of dengue residing in endemic areas [10]. Vector control based on the reduction of larval habitats and space-spraying of insecticides remains the most common approach to reduce the incidence of dengue in the general population [11, 12].

Pyrethroids are insecticides most commonly used to control adult *Ae. aegypti* [13]. Knockdown resistance (*kdr*) is one of the most important mechanisms of resistance to pesticides documented globally in a large number of arthropods of medical importance [14]. Pyrethroids (e.g., deltamethrin, permethrin) and organochlorines (e.g., dichlorodiphenyltrichloroethane, DDT) are insecticides that trigger the *kdr* phenotype [15]. Mutations in the gene encoding voltage-gated sodium channels (VGSC) are associated with the *kdr* phenotype. Overall, 12 non-synonymous mutations varying in frequency, geographical distribution, and impact on resistance phenotype, have so far been detected across the world, of which six (V410L, L982W + F1534C, S989P, I1011M/V, V1016G/I, and F1534C) have been demonstrated to be associated with pyrethroid resistance in *Ae. aegypti* [13, 16]. The mutations S989P, I1011M or V, V1016G or I, and F1534C occur within domains II and III of VGSC. Recent studies have shown that three of these mutations, 1016G, 1016I, and 1534C, are widely distributed and detected in pyrethroid-resistant populations of *Aedes* mosquitoes in several African countries, including Cameroon, Côte d’Ivoire, Burkina Faso, Cape Verde, and Ghana [17–22].

Historically, the first vector control program targeting malaria vector in Mauritania was implemented in the early 1990s. It was first based on the promotion of the use of insecticide-treated nets (ITNs), and then a few years later, long-lasting insecticide-treated nets (LLINs) were introduced to replace the conventional ITNs. Mass distribution of pyrethroid-impregnated (deltamethrin and permethrin) mosquito nets has been the main vector control intervention since 1997. Indoor residual spraying and larval control are also part of the priorities recently identified in the national malaria control policy, but they have not yet received the necessary funding for their implementation. Studies assessing the impact of LLINs in reducing the incidence of malaria and other vector-borne diseases in the country are lacking.

Among mosquitoes of medical importance, the phenotypic and molecular basis of resistance to insecticides has been studied in the malaria vector *Anopheles arabiensis* but not in *Ae. aegypti* in Mauritania [23, 24]. The present study assessed the frequencies of six point mutations known to confer pyrethroid resistance in *Ae. aegypti* collected in Nouakchott in 2017–2018, i.e., 3 years after the first documented dengue outbreak following the detection of the arboviral vector in the city [2, 4], with the objective of establishing baseline data for future arbovirus surveillance and vector control strategies.

## Methods

### Study sites

The study was part of the entomological surveillance of malaria and dengue vectors in Nouakchott, the capital city of Mauritania. It was carried out in the districts of Dar Naim and Teyarett located in northern and northeastern areas of Nouakchott. A detailed description of the study sites was presented in our earlier works [4, 23, 25]. In Teyarett and Dar Naim, malaria is known to be endemic, and several epidemiological, parasitological, and entomological studies on malaria in Nouakchott have been conducted in these two districts [25–28]. Teyarett was also the epicenter of the first dengue outbreak that occurred in Mauritania in 2014. Dengue cases have been reported from the district of Dar Naim as well. Based on clinical reports of dengue fever occurring in Nouakchott, entomological monitoring of *Ae. aegypti*, the most probable vector of dengue, was initiated in these two districts 3 years (i.e., from 2017) after the first detection of this mosquito species in Nouakchott.

### Mosquito sampling

A battery-powered Prokopack aspirator (John W. Hock Company, Gainsville, FL, USA) was used to collect indoor resting culicid fauna. The collection was carried out during the morning (between 07:00 and 09:00) for one to four consecutive days per month in each site (five houses/site and two rooms per house). Overall, mosquitoes were collected from March to June 2017 and during the month of September 2018. Upon collection, mosquitoes were euthanized by placing them in a −20 °C freezer. They were then sorted by species and sex using appropriate taxonomic keys [29]. Female *Ae. aegypti* were further classified according to abdominal status (unfed, fed, semi-gravid, or gravid), then stored at −80 °C until use for *kdr* genotyping.

### Genotyping *kdr*

*Aedes aegypti* specimens were placed in microtubes with 5-mm diameter stainless steel beads for use with the TissueLyser system (Qiagen S.A.S., Courtaboeuf, France) and 205 µl of lysis buffer (25 µl of proteinase K and 180 µl of T1 buffer in the NucleoSpin^®^ 96 tissue core kit). Specimens were crushed in the TissueLyser II (Qiagen S.A.S., Courtaboeuf, France) with three cycles of 1 min at a frequency of 30/s separated by 20 s of pause time. Samples were incubated at 70 °C for 1 h. Mosquito DNA was extracted using the NucleoSpin^®^ 96 tissue core kit (Macherey Nagel AG, Oensingen, Switzerland) following the manufacturer’s instructions.

Two fragments of the *vgsc* gene were amplified in two separate polymerase chain reactions (PCR) using primer pairs designed with Geneious Prime software version 2022.2.2 (https://www.geneious.com). In the reaction of the first fragment, primer pairs *vgsc* 20–21 F (forward primer, 5′-CTGCCACGGTGGAACTTCA-3′) and *vgsc* 20–21 R (reverse primer, 5′-TTGTTCGTTTCGTTGTCGGC-3′; 0.25 µM each) were used to genotype L982W, S989P, I1011M/G, and V1016G/I codons. For the second fragment, primer pairs *vgsc* 28 F (forward primer, 5′-GTGGGAAAGCAGCCGATTC-3′) and *vgsc* 28 R (reverse primer, 5′-CCTAGGCCGTGGAATAGCTT-3′; 0.375 µM each) were used to genotype F1534C codon. The primer pairs and 3 µl of eluted DNA template were added to PCR master mix containing DreamTaq DNA polymerase, buffer, 2 mM MgCl_2_, and deoxyribose nucleoside triphosphate (dNTP) (Thermo Fisher DreamTaq™ Green PCR Master Mix; Thermo Fisher Scientific, Illkirch, France). The thermal cycler (T1 Biometra, Thermo Fisher Scientific, Illkirch, France) was programmed as follows: initial step of 95 °C for 5 min, then 35 cycles of 95 °C for 1 min, 59 °C (annealing temperature) for 1 min, and 72 °C for 1 min, followed by an extension at 72 °C for 10 min. The quality of PCR products was checked by visualizing under ultraviolet light the presence of a band of expected sizes (304 base pairs [bp] and 241 bp for the first and second *vgsc* gene fragments, respectively) after agarose gel electrophoresis.

Purification of PCR products and sequencing were outsourced to Biofidal Themis (Vaulx-en-Velin, France). Each fragment was sequenced from both 5′ and 3′ ends. The sequences were aligned and compared with those of GenBank accession nos. MK977832.1 and MF794973.1 using Molecular Evolutionary Genetics Analysis (MEGA, version 10) software.

### Statistical analysis

Data were entered into an Excel spreadsheet (Microsoft Office Excel 2007, Microsoft Corporation, Redmond, WA, USA). The proportions of unfed, fed, and gravid resting females were compared using Fisher’s exact test. Hardy–Weinberg equilibrium for observed genotype frequencies for each *kdr* mutation was calculated using the Chi-square test. Statistical significance was set at *P* < 0.05. All data were analyzed using Excel software or MedCalc^®^ statistical software version 20.115 (MedCalc Software Ltd, Ostend, Belgium; https://www.medcalc.org; 2022).

## Results

A total of 100 *Ae. aegypti* female adults were collected in 2017 (*n* = 50) and 2018 (*n* = 50) during the entomological surveillance of *Anopheles* and *Aedes* mosquitoes in Teyarett and Dar Naim districts of Nouakchott.

The abdominal status of the resting female mosquitoes is summarized in Table 1. Sixty (60%) resting female *Aedes* mosquitoes were unfed, 28 (28%) were gravid, and 12 (12%) were freshly blood-fed.

**Table 1:** Abdominal status of resting female *Aedes aegypti* mosquitoes collected in Nouakchott, Mauritania

Abdominal status	Number	Frequency	*P*-value^b^
Unfed	60	0.60	0.005
Freshly blood-fed	12	0.12	
Gravid^a^	28	0.28	
Total	100	1.00	

^a^Including half-gravid female

^b^Comparison of frequencies between unfed and gravid female *Ae. aegypti* mosquitoes

### *Kdr* genotyping

Table 2 presents the results of *vgsc* genotyping. All successfully genotyped mosquitoes were homozygous wild-type (i.e., monomorphic) for the *kdr* codons 982 (*n* = 81) and 1011 (*n* = 85). The three remaining *kdr* point mutations, 989P, 1016G, and 1534C, were found among the investigated *Aedes* mosquitoes with allelic frequencies of 0.43, 0.44, and 0.55, respectively. There were no statistical differences in the frequencies of *kdr* point mutations in *Aedes* mosquitoes collected in 2017 compared with those collected in 2018 (*P* = 0.55, Fisher’s exact test).

**Table 2:** Allelic and genotypic frequencies of *kdr* mutations in female *Aedes aegypti* mosquitoes, Nouakchott, Mauritania

Year	Number	*kdr* allele	*N* (%)				Frequency^a^	*χ*^2^ HWE	*P*-value
			SS	SR	RR	UI			
2017	50	L982W	37 (74)	0 (0)	0 (0)	13 (26)	0	NA	NA
		S989P	18 (36)	18 (36)	5 (10)	9 (18)	0.34	0.02	0.87
		I1011M	41 (82)	0 (0)	0 (0)	9 (18)	0	NA	NA
		V1016G	17 (34)	16 (32)	5 (10)	12 (24)	0.34	0.16	0.69
		F1534C	6 (12)	22 (44)	18 (36)	4 (8)	0.63	1.44	0.23
2018	50	L982W	44 (88)	0 (0)	0 (0)	6 (12)	0	NA	NA
		S989P	10 (20)	23 (46)	11 (22)	6 (12)	0.51	0.09	0.76
		I1011M	44 (88)	0 (0)	0 (0)	6 (12)	0	NA	NA
		V1016G	10 (20)	23 (46)	12 (24)	5 (10)	0.52	0.03	0.87
		F1534C	12 (24)	26 (52)	9 (18)	3 (6)	0.74	0.58	0.45
2017–2018	100	L982W	81 (81)	0 (0)	0 (0)	19 (19)	0	NA	NA
		S989P	28 (28)	41 (41)	16 (16)	15 (15)	0.43	0.02	0.89
		I1011M	85 (85)	0 (0)	0 (0)	15 (15)	0	NA	NA
		V1016G	27 (27)	39 (39)	17 (17)	17 (17)	0.44	0.18	0.67
		F1534C	18 (18)	48 (48)	27 (27)	7 (7)	0.55	0.16	0.69

S, susceptible; R, resistant; UI, uninterpretable; *kdr*, knockdown resistance; HWE, Hardy–Weinberg equilibrium at 5% significance level; NA, not applicable

^a^Frequency of mutant allele

Genotype distribution did not deviate from Hardy–Weinberg equilibrium at any of the three codons (i.e., S989P, V1016G, and F1534C). The most frequent genotypes detected were heterozygous 1534FC, 989SP, and 1016VG, with frequency of 48%, 41%, and 39%, respectively. The homozygous wild-type 1534FF and the homozygous mutant-type 1016GG and 989PP had the lowest frequencies (18%, 17%, and 16%, respectively).

### Haplotypes

**Fig. 1:**
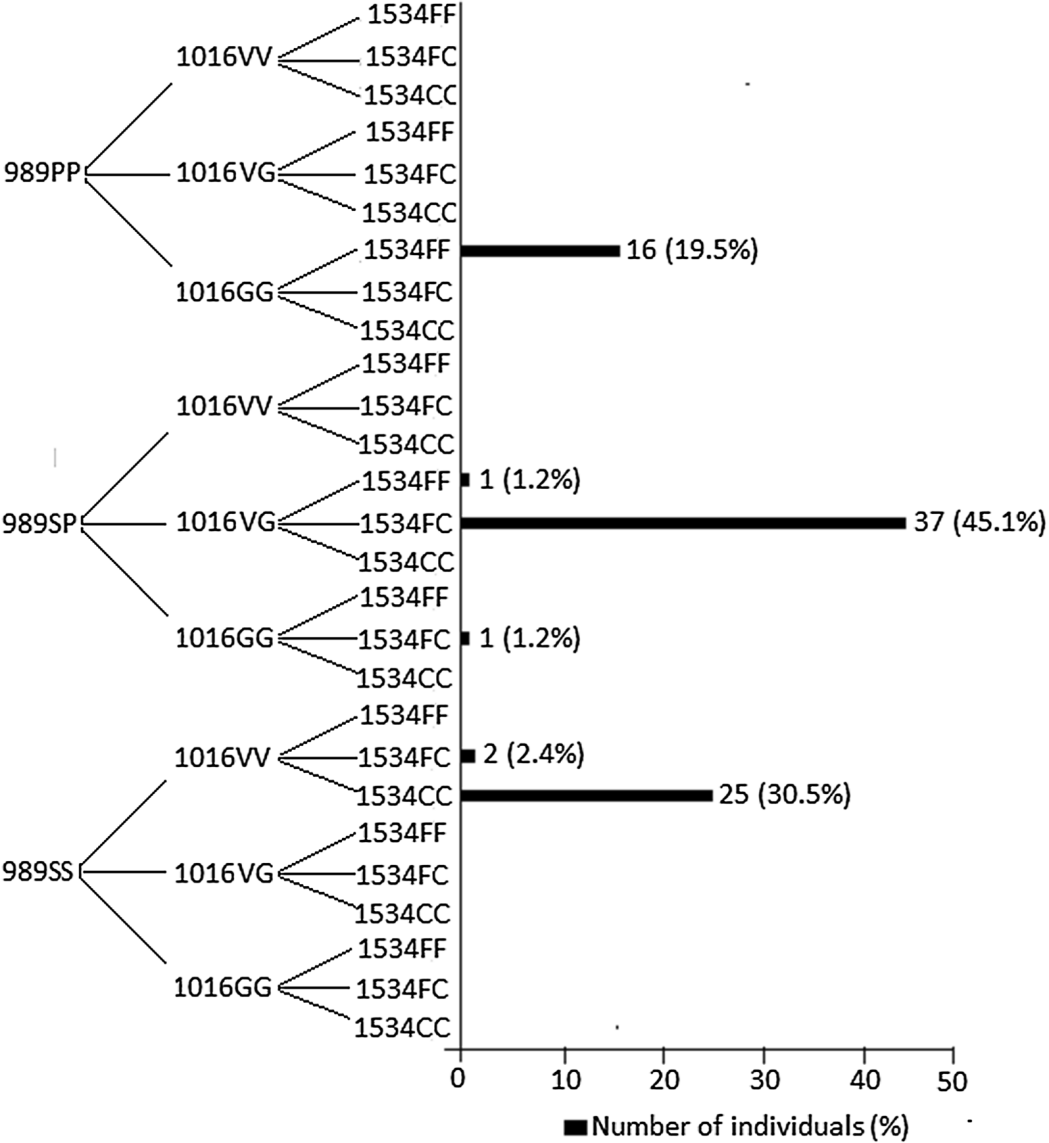
The combinations of *kdr* point mutations S989P, V1016G, and F1534C in adult female *Aedes aegypti* mosquitoes in Nouakchott, Mauritania

The combination of *kdr* mutations in three polymorphic codons (i.e., S989P, V1016G, and F1534C) was analyzed, and the results are presented in Fig. 1. Six (22.2%) of 27 possible allelic combinations were observed among *Aedes* mosquitoes. Of 82 mosquitoes that were successfully genotyped, 37 (45.1%) were heterozygous for three mutations (i.e., 989SP/1016VG/1534FC), 25 (30.5%) were single-mutant for the 1534C allele (989SS/1016VV/1534CC), and 16 (19.5%) were double-mutant with 989P and 1016G mutations (989PP/1016GG/1534FF). None were homozygous wild-type (989SS/1016VV/1534FF) or homozygous mutant-type (989PP/1016GG/1534CC).

## Discussion

The present study was conducted to determine the frequencies of *kdr* mutations in adult female *Ae. aegypti* collected a few years after the establishment of the arbovirus vector in Nouakchott. The collection of indoor resting female mosquitoes showed that a high proportion of *Aedes* mosquitoes were unfed. Studies conducted in Asia and Latin America showed that *Ae. aegypti* populations are typically endophagic and endophilic, resting and feeding in human habitations [30, 31]; in West Africa, however, studies revealed a broader range of resting behaviors [32–34]. For instance, studies conducted in Niger showed endophilic and endophagic behavior of *Ae. aegypti* populations [32], while in Ghana and Senegal, *Ae. aegypti* populations were collected mostly outdoors rather than indoors [33, 34]. In the present study, *Ae. aegypti* exhibited a similar behavior as that reported from Senegal and Ghana, but given the low number of unfed mosquitoes, a solid conclusion cannot be drawn at present. However, this finding is important for vector control strategies and points to the need for more in-depth studies on the bioecology of *Ae. aegypti* in Mauritania.

Furthermore, we analyzed the prevalence of *kdr* mutations in the *vgsc* gene of individual *Ae. aegypti* mosquitoes in Nouakchott. Two major African-type *kdr* mutations strongly linked to pyrethroid resistance, 1016G and 1534C, were found in *Ae. aegypti* analyzed in this study with high allelic frequencies: 1016G (0.44) and 1534C (0.55). For the first time, the Asian-type *kdr* mutation linked to pyrethroid resistance 989P [35] was found in Mauritanian *Ae. aegypti* with high allelic frequency (0.43). More interestingly, a high proportion (45.1%) of *Aedes* mosquitoes harbored the triple mutations 989SP/1016VG/1534FC with the heterozygous status, suggesting a potentially high level of resistance to pyrethroids. Similar findings were reported from Myanmar [36]. However, in a study conducted in Senegal [37], no mutations in the *vgsc* gene associated with pyrethroid resistance were found despite the phenotypic resistance to pyrethroids observed in *Ae. aegypti* populations. In that study, insecticide resistance of *Ae. aegypti* population was found to be associated with a remarkably high (20- to 70-fold) overexpression of the major detoxification genes, suggesting that insecticide resistance in investigated *Ae. aegypti* specimens was likely to be mediated by metabolic mechanisms. In another study conducted in Cape Verde [38], many of the mutations commonly associated with insecticide resistance, including V1016G/I, S989P, and F1534C, were detected in *Ae. aegypti* screened samples, but insecticide resistance/susceptibility phenotype was not determined in that study. However, in two West African countries, Ghana and Burkina Faso, two of the mutations detected in the present study (F1534C and V1016G) were found at a high frequency among pyrethroid-resistant *Ae. aegypti* populations [19, 20, 39, 40]. These findings on high frequencies of *kdr* mutations are not surprising since pyrethroids remain the most commonly used class of insecticides in vector control worldwide [41].

Studies on *kdr* mutations in *Ae. aegypti* in West Africa are still scarce. However, point mutations reported here, in particular F1534C and V1016G, have been shown to play an important role in conferring insecticide resistance to local *Aedes* mosquitoes. Indeed, since its first detection in *Ae. aegypti* populations from Ghana [42], *Aedes* mosquitoes with the F1534C mutation have been spreading in Africa. This mutation has since then been reported from Côte d'Ivoire [18], Cameroon [43], Angola, and Cape Verde [20]. In Burkina Faso, the F1534C substitution was found to be almost fixed in the populations of *Ae. aegypti* in the capital city, Ouagadougou [39]. Concomitant presence of these point mutations was also reported to strongly increase the level of pyrethroid resistance in individual mosquitoes [44]. In our study, high proportions of female *Aedes* mosquitoes displayed double or triple mutations, possibly implying a high level of pyrethroid resistance [45].

In our study, we observed that homozygous wild-type genotypes at codon 1534 co-occurred with homozygous mutants at 989P and 1016G, and vice versa. This finding makes it more important to look for the other mutations present in domains II and III of VGSC in *Aedes*. In Asia, but not yet in Africa, novel mutations have been reported to be associated with pyrethroid resistance: A1007G [46], T1520I [47],  and F1534L [48]. In our study, we did not observe F1534L and A1007G. Future studies in Africa should include monitoring of these mutations.

In Mauritania, the first report of *Ae. aegypti* in Nouakchott dates back to 2014 [4]. However, this situation alone cannot explain the potentially high frequencies of *kdr* mutations observed among *Ae. aegypti* mosquitoes collected 3 years later in two districts of Nouakchott. Therefore, it can be hypothesized that *Aedes* mosquitoes harboring different *kdr* point mutations may have been introduced in Nouakchott from other sites or countries, followed by cross-mating between them, resulting in highly heterogeneous mosquito populations. However, the unknown impact and contribution of vector control campaigns through aerial spraying of insecticide carried out in recent years to reduce mosquito nuisance, particularly in Nouakchott, cannot be overlooked [28].

With the rapid and disorganized urbanization process that Nouakchott has been experiencing over the past 20 years, along with the rapidly growing human population and massive importation of second-hand tires resulting from the growing demand of used cars imported from Europe, Nouakchott offers an environment that is conducive to maintaining the habitats of *Ae. aegypti*. Indeed, studies from Kenya and Tanzania showed that tires provide good breeding sites for *Aedes* mosquitoes and are responsible for producing > 30% of immatures collected from larval habitats in urban areas [49, 50]. The alarming findings of the present study call for an effective vector control strategy based on reinforced entomological surveillance, integrated approaches to control both larval and adult stages of *Aedes* mosquitoes, and fully engaged community-led actions.

The main limitations of the present study are that the insecticide resistance/susceptibility phenotype of genotyped mosquitoes was not assessed during the study to correlate the observed genotypes with their corresponding insecticide resistance phenotypes and that the recently detected V410L *kdr* mutation associated with high levels of resistance to both types I and II of pyrethroids was not searched [51]. Another limitation was that our sample size was small.

## Conclusions

This is the first report on the presence of *kdr* mutations in *Ae. aegypti* from Mauritania since this mosquito species was established in Nouakchott. The findings of the present study are alarming because they predict a high level of resistance to pyrethroids in the *Ae. aegypti* population found in the capital city. Further studies are urgently needed, in particular phenotypic characterization of insecticide resistance using the standardized test in parallel with genotyping more recent collection of *Ae. aegypti* mosquitoes. Furthermore, as insecticide resistance negatively impacts vector control, regular surveillance of the resistance/susceptibility status of the mosquito vector population should be implemented, with a primary focus on the chemical insecticides used in vector control in Mauritania.

## Data Availability

All data generated or analyzed during this study are included in this published article.

## Abbreviations

bp: Base pairs
DDT: Dichlorodiphenyltrichloroethane
dNTP: Deoxyribose nucleoside triphosphate
ITN: Insecticide-treated nets
*kdr*: Knockdown resistance
LLIN: Long-lasting insecticide-treated nets
MEGA: Molecular Evolutionary Genetics Analysis
PCR: Polymerase chain reaction
VGSC: Voltage-gated sodium channel
